# Lip‐closing strength in children is enhanced by lip and facial muscle training

**DOI:** 10.1002/cre2.490

**Published:** 2021-09-09

**Authors:** Yukiko Nogami, Issei Saitoh, Emi Inada, Daisuke Murakami, Yoko Iwase, Naoko Kubota, Yuki Nakamura, Kuniko Nakakura‐Ohshima, Ayako Suzuki, Youichi Yamasaki, Haruaki Hayasaki, Yasutaka Kaihara

**Affiliations:** ^1^ Division of Pediatric Dentistry, Graduate School of Medical and Dental Science Niigata University Niigata Japan; ^2^ Department of Pediatric Dentistry Asahi University School of Dentistry Mizuho Japan; ^3^ Department of Pediatric Dentistry Kagoshima University Graduate School of Medical and Dental Sciences Kagoshima Japan; ^4^ Department of Dentistry for the Disability and Oral Health Asahi University School of Dentistry Gifu Japan; ^5^ Department of Dental Hygiene Ogaki Women's College Ogaki Japan

**Keywords:** incompetent lip seal, lip‐closing strength, lip‐closing training, malocclusion

## Abstract

**Objectives:**

Weakening of lip‐closing strength (LCS) associated with an incompetent lip seal (ILS) may affect the oral balance between the lip and tongue pressures. The purpose of this study was to evaluate the effects of lip‐closing training in children with lower LCS and/or abnormal habits across different age groups and to compare its effects on increasing LCS in children with malocclusion and/or oral habits.

**Material and Methods:**

Lip‐closing training was performed by 154 Japanese children aged 3–12 years using a specialized training device at home for 3 months. Children with oral habits and/or exhibiting less than standard LCS were included. LCS was measured using a digital strain force gauge at a dental clinic at the beginning (T0) and after each month (after 3 months: T3).

**Results:**

Children had higher LCS responses after lip‐closing training. The first month of lip‐closing training was more effective than the subsequent months. With lip‐closing training, the LCS increased from an average of 6.2 N (T0) to 11.4 N (T3) in Group I, 7.9 N (T0) to 12.8 N (T3) in Group II, and 6.8 N to 11.4 N in Group III. Anterior cross bite, including reverse bite, open bite, and tongue thrusting, significantly reduced training effects.

**Conclusion:**

Our findings showed that lower LCS in children with ILS resulted in greater responses to lip‐closing training in a short period, but oral dysfunction, such as abnormal habits, inhibited the positive effects of training. Our results suggest that less detrimental effects of malocclusion and abnormal oral habits lip‐closing training enhances LCS in younger children.

## 1 INTRODUCTION

Incompetent lip seal (ILS) is defined as insufficiency in maintaining the lips together, which results from a dysfunction in the orofacial area (Saitoh et al., 2018). Keeping the mouth closed at rest is difficult, and the hypertonic muscular contraction associated with forceful closing is visible. During childhood, the prevalence of ILS is at least 30% and increases significantly with age (de Menezes et al., 2007; Nogami et al., 2021; Yata et al., 2001). ILS causes developmental deficiencies in craniofacial growth, tooth eruption, and alignment, swallowing, and temporomandibular joint function (Drevensek et al., 2005; Gulati et al., 1998). ILS affects the oral balance between the lip and tongue pressures and can result in labial tipping of the maxillary anterior teeth and narrowing the maxillary dental arch. Bresolin et al. reported that children with severe open mouth postures present with significantly reduced growth of the maxillary dental arch (Bresolin et al., 1984). Gross et al. also reported that a prolonged open mouth posture during childhood results in a narrow maxillary arch and longer facial height (Gross et al., 1990). Moreover, Inada et al. reported that ILS affects facial soft tissue form, exemplified by nasal prominence, sagittal facial convexity, and acicular lips in early childhood (Inada et al., 2019). ILS can also cause common clinical manifestations and symptoms of airway obstruction, such as mouth breathing, allergic disorders, asthma, sore throat, and rhinitis; in addition, these clinical manifestations can induce ILS (Diouf et al., 2018; Galvez & Methenitou, 1989; Hu et al., 2018). ILS is also associated with physical and mental symptoms such as stiff shoulders, lack of sleep, and chronic fatigue (Suzuki et al., 2017; Takada et al., 2018). For these reasons, ILS should be treated at an early stage before the irreversible effects of continuous ILS on orofacial hard and soft tissues manifest.

Lip‐closing training has been reported previously. For instance, Ambrosio et al. demonstrated that the electromyographic activity of the upper lip in mouth breathing adults with Angle Class II Division 1 malocclusion differs from that in nasal breathing adults (Ambrosio et al., 2009). Several reports have emphasized the use of electromyography (EMG) findings to support the development of a lip training program for adults with lip dysfunction. Yoshizawa et al. investigated the differences in EMG findings of the orbicularis oris muscles between adult subjects with lip incompetence and competence. They suggested that standardized lip training could improve the EMG activity while the lips were closed (Yoshizawa et al., 2018). In another study, Busanello‐Stella et al., using an EMG signal, reported that mouth‐breathing children felt fatigued more easily in the orbicularis oris muscle compared with the nasal‐breathing children (Busanello‐Stella et al., 2015). The investigation of myofunctional treatment during growing periods was limited to several reports of children with neuromuscular dysfunction, such as a lip training method for patients with intellectual disability or lip deformation (da Costa et al., 2018; Pinheiro et al., 2018; Saccomanno et al., 2018). To date, limited studies have analyzed the effects of oral functional training during developmental periods (Quinzi et al., 2020).

Several reports indicate that children with ILS have lower lip‐closing strength (LCS) (Inada et al., 2019; Lambrechts et al., 2010; Saitoh et al., 2017; Saitoh et al., 2018). Therefore, lip‐closing training involving the facial muscles at an early stage is considered useful in improving the LCS. The increase in LCS in children is associated with age, and it was hypothesized that the effects of training might differ according to age.

The purpose of this study was to evaluate the effects of lip‐closing training in children with lower LCS and/or abnormal habits across different age groups. This study was conducted with the expectation that lip‐closing training would increase the LCS and evaluate LCS changes in children with malocclusion and/or abnormal oral habits.

## 2 MATERIALS AND METHODS

### 2.1 Study design and participants

In this study, 154 Japanese children (74 boys and 80 girls) aged 3–12 years (Table 1), who visited 26 dental clinics specializing in pediatric dentistry for oral examinations with regular follow‐ups, were included. The sample size was estimated using G*Power for Windows (version 3.1.9.4, Franz Faul, University Kiel, Germany) and was calculated for 90% power, *α* = 0.05, and effect size = 0.3. Children with abnormal oral habits or a less than standard LCS were included. The LCS was measured using a dedicated device, and standard values were determined based on published data (Saitoh et al., 2017). Oral habits were diagnosed by pediatric dentists. ILS was defined as the inability of the child to keep the mouth closed at rest, determined by visual judgment and parent interviews. Exclusion criteria included severe disease in the maxillofacial region, severe dental caries, or need for or history of restorative treatment. Pediatric dentists confirmed abnormal oral habits and malocclusion and the absence of any severe jaw and oral function problems in the participants. The criteria for each age group were based on our study's assessment that increases in the growth LCS occurred differently with age (Saitoh et al., 2017). Age groups were established by grouping ages that indicated a similar pattern of ILS increase. The participants were classified into three groups according to age (mean [SD]: Group I: 5.4 [1.0] years, Group II: 8.3 [0.8] years, and Group III: 10.8 [0.6] years) (Table 1). This study received ethical approval from the Graduate School of Medical and Dental Sciences, Kagoshima University, in Kagoshima (approval number: 669). Written informed consents were obtained from all children's parents according to the principles of the Helsinki Declaration.

**Table 1:** Participant classification

Classification	Age (years)	Average age and months (SD)	*n*	Sex	
				Boy	Girl
Group I	3–6	5.4 (1.0)	66	31	35
Group II	7–9	8.3 (0.8)	68	35	33
Group III	10–12	10.8 (0.6)	20	8	12
Total			154	74	80

Abbreviation: SD; standard deviation.

### 2.2 Measurement of lip‐closing strength and lip‐closing training

**Figure 1:**
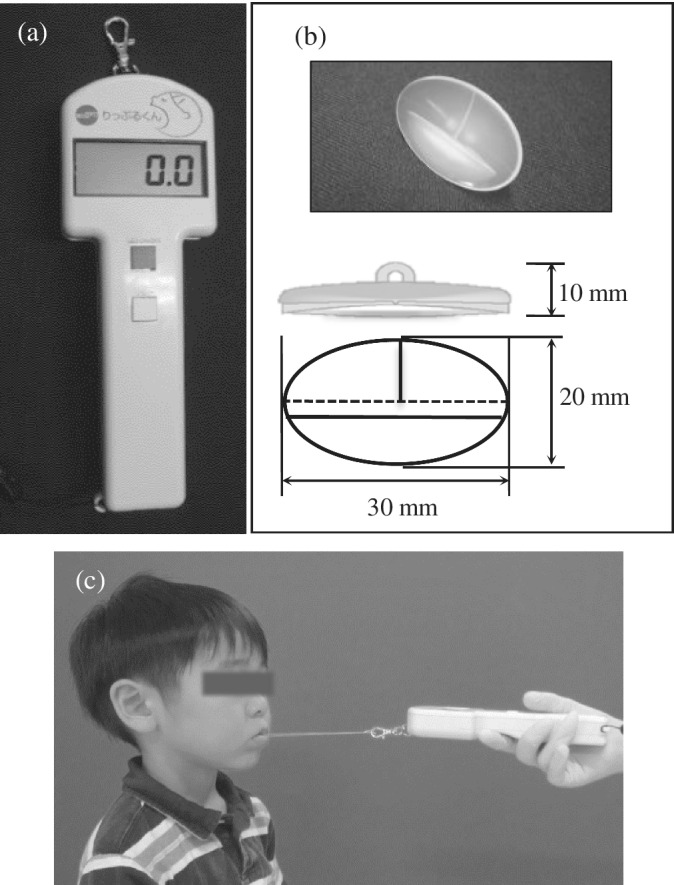
Measurement of lip‐closing strength. (a) Digital strain force gauge (Lipplekun®), (b) Lipple button®, and (c) Lipple button® attached to Lipplekun® by dental floss was inserted into the upper and lower labial vestibules and pulled outwards, parallel to the floor

LCS was measured using a digital strain force gauge (Lipplekun®, SHOFU Inc., Kyoto, Japan) at each dental clinic (Figure 1), as previously described (Saitoh et al., 2017). LCS was measured as the force resisted by the device while pulling the tightly closed lips. The measurement instruments used in this study are employed by numerous dental clinics and are frequently used in measurement techniques. The average of three measured numerical values was used.

**Figure 2:**
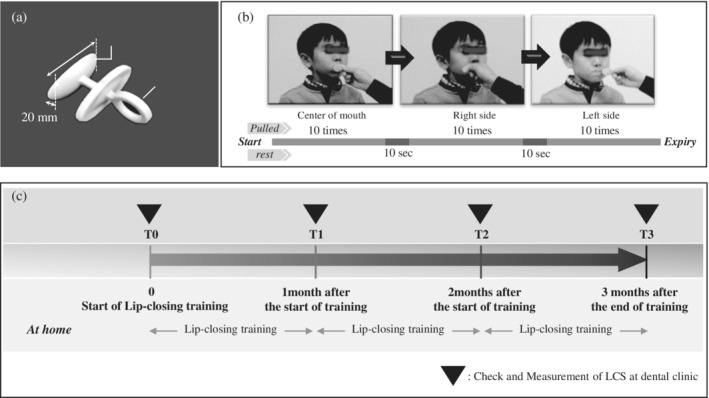
Lip‐closing training protocol (a,b) and lip‐closing training timeline (c) at home. (a) Lipple‐trainer® consisting of a lip holder, stopper, and a pull ring. The lip holder is inserted into the oral vestibule. When the parent pulls the pull ring, the participant holds the lip holder between the lips. (b) Lip‐closing training protocol: parents pull the Lipple‐trainer® 10 times at the center, right, and left, and rest for 10 s between each site. (c) Participants visited the clinic for measurement of lip‐closing strength (LCS). The initial visit was denoted T0, second visit after 1 month as T1, third visit after 2 months as T2, and fourth visit after 3 months as T3. Dentists or dental hygienists lectured participants on lip‐closing training in the clinic. LCS measurement and lip‐closing training were performed at T0, T1, T2, and T3 in the clinic. The participants trained their lips and facial muscles using the Lipple‐trainer® every day at home

The training method was presented to the dentists and dental hygienists in each dental clinic through documents and videos. After mastering the technique, they instructed the participants. At the first visit, dentists and dental hygienists provided instructions regarding lip‐closing training to participants and their parents at the clinics, and after sufficient practice, the participants trained at home. The training was performed using the training device Lipple‐trainer® (SHOFU Inc., Kyoto, Japan) (Figure 2a). The Lipple‐trainer® comprises a lip holder (45 mm × 20 mm), stopper, and a pull ring the same size used for all participants. The Lipple‐trainer® is made of plastic with the lip holder and pulls ring in one piece and the pull ring in the middle line. Traumatic injuries to the mouth can be prevented by the stopper even if the device slips during training. The subjects' training was based on the instructions for the use of the Lipple‐ trainer®. The Lipple‐holder was inserted into the upper and lower labial vestibules and held by pressure from the lip muscles. The participants resisted the force of pulling the pull ring behind closed lips by slowly activating the orbicularis oris muscle. Participants were seated in chairs with natural head postures such that their eye–ear planes were parallel to the floor. At home, the participants' parents or the participant himself/herself pulled the Lipple‐trainer® 10 times repeatedly, increasing the pulling pressure parallel to the floor. In the case of younger participants, the participants' parents pulled the Lipple‐trainer®. A set of lip‐closing training using the Lipple‐trainer® was performed and repeated at each of three positions (center of the lip and left and right corners of the lips) every day; there was a 10‐s rest during each training session (Figure 2b).

The study timeline is shown in Figure 2c. The timeline shows lip‐closing training at home for 3 months and four measurements of LCS in the clinic. Participants visited a dental clinic to evaluate the response to training and for LCS measurements once a month for 3 months. At each visit to the dental office, the participant's adherence to the training protocol was confirmed by verifying the correct training methods. The first visit to the clinic for training was defined as T0, second visit after 1 month as T1, third visit after 2 months as T2, and fourth visit after 3 months as T3. LCS measurements and lip‐closing training were performed at T0, T1, T2, and T3 in the clinic, using T0 as the baseline.

### 2.3 Statistical analysis

The LCS data were analyzed using the Shapiro–Wilk test and one‐way repeated measures analysis of variance with post‐hoc multiple comparisons using a paired *t*‐test with Bonferroni correction for each group (Group I, Group II, and Group III). The three groups were formed based on participant age. Data for effects of lip‐closing training on LCS were analyzed for normal distribution using the Shapiro–Wilk test. Equality of variances was checked using Levene's test. Student's *t*‐test (normally distributed values) or Mann–Whitney *U* test (non‐normally distributed values) was performed for bivariate comparisons. Statistical analyses were performed using IBM SPSS Statistics for Windows (version 20; SPSS, Inc., Tokyo, Japan). The significance level was set at *p* < 0.05.

## 3 RESULTS

Data for 207 participants were obtained from 26 pediatric dental clinics according to the inclusion criteria of this study. Minor errors in the number of times LCS measurements or survey responses led to the exclusion of 49 participants (24%). Four additional participants (2%) dropped out during the training period. Therefore, the final number of participants in this study was 154. The Shapiro–Wilk test confirmed a normal data distribution (*p* > 0.05). The analysis of variance test showed significant differences (*p* < 0.001), and post‐hoc multiple comparisons using paired *t*‐test with Bonferroni correction confirmed significant differences between time points within the three groups.

**Figure 3:**
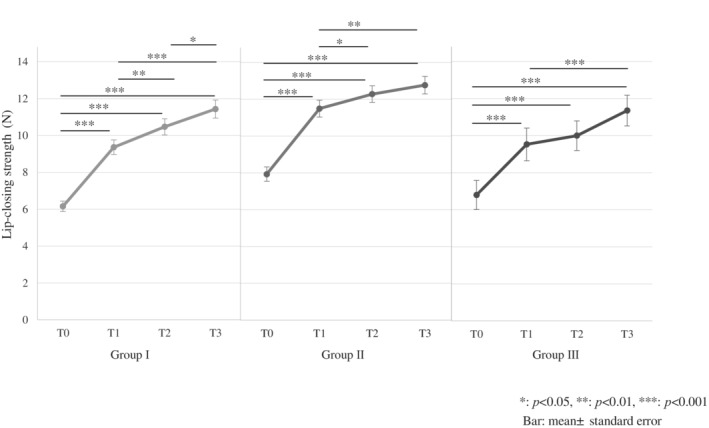
Lip‐closing strength (LCS) after each month. LCS significantly increased after lip‐closing training for 3 months in each group

**Figure 4:**
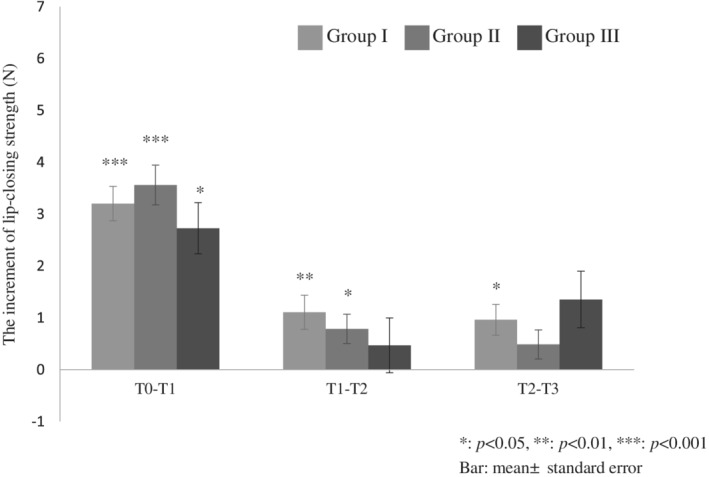
Increase in lip‐closing strength after lip‐closing training for 1 month

LCS data before and after lip‐closing training for each month is shown in Figure 3. Lip‐closing training for 3 months significantly enhanced the LCS in all groups (Group I: mean 6.2–11.4 N, Group II: mean 7.9–12.8 N, and Group III: mean 6.8–11.4 N). A multiple comparison test revealed significant differences in Groups I, II, and III between T0–T1, T0–T2, and T0–T3 (*p* < 0.001 for all three groups), in Groups I (*p* = 0.009) and II (*p* = 0.041) between T1 and T2, in Groups I (*p* < 0.001), II (*p* = 0.002), and III (*p* < 0.001) between T1 and T3, and in Group I (*p* = 0.011) between T2 and T3 (Figure 3). All other differences, that is, in Groups II and III between T2 and T3 (*p* = 0.511 for both groups) and in Group III between T1 and T2 (*p* = 1.0), were not significant. In Group I, the differences of LCS between each interval for all time points were significant; however, as age increased, the number of time points with significant differences decreased. Therefore, the variation in ILS values for each month was focused. After 1 month of lip‐closing training, significant increments were observed (T0–T1) in all groups (Figure 4). However, significant increases in LCS after 2 months of lip‐closing training were observed in Groups I and II, whereas significant increases in LCS after 3 months of lip‐closing training were observed only in Group I (Figure 4).

**Figure 5:**
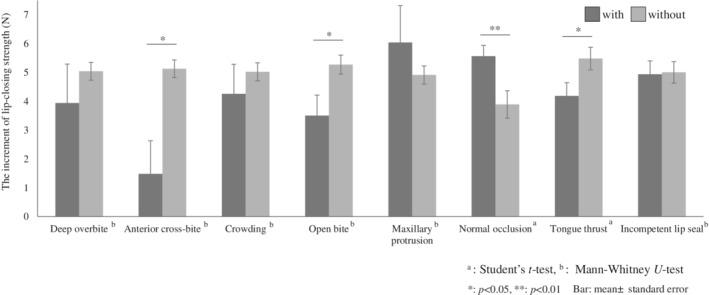
Malocclusion or abnormal habits affect the increases in lip‐closing strength after lip‐closing training for 3 months

Finally, the effect of the presence of malocclusion and/or abnormal oral habits on the increase in LCS after 3 months of lip‐closing training was investigated (Figure 5). Student's *t*‐test or Mann–Whitney *U* test was used to compare malocclusion and/or abnormal oral habits between the two groups. Anterior cross bite, including reverse bite (4.5%) and open bite (16.2%) reduced the effects of lip‐closing training, whereas the presence of normal occlusion significantly enhanced the effects. Tongue thrusting (34.4%) significantly reduced training effects, but ILS (24.7%) did not affect the training.

## 4 DISCUSSION

This study among 154 Japanese children (3–12 years old) aimed to evaluate lip‐closing training effects in children with ILS and/or abnormal habits across different age groups and compare its effect on increasing LCS in children with malocclusion and/or oral habits. Abnormal tongue posture is reported to be the cause of open bite and adversely affects the lack of anterior oral seal (Fränkel, 1980). Therefore, abnormal tongue posture or open bite may have interfered with the effectiveness of the training. There were significant differences in Groups I and II between T0–T1 and T0–T2 and in Groups I and III between T0–T2 and T0–T3. Anterior cross bite, including reverse bite, open bite, and tongue thrusting, reduced the effects of lip‐closing training.

The digital strain force gauge Lipplekun® was used to measure LCS, as reported previously (Saitoh et al., 2017). Lipplekun®, which is approved by the Ministry of Health, Labour and Welfare in Japan, has a broad measurement spectrum ranging from younger children to older adults. Other studies have used the device in adults. Our study used the device and performed measurements in children. The values of measurements taken in younger children in this study were similar or higher than those found in studies that used other lip‐closing methods (Fukami et al., 2010; Lambrechts et al., 2010; Yamanaka et al., 2011). This could be due to the lack of understanding and concentration in younger children.

Kaede et al. demonstrated that lip‐closing training enhances LCS in healthy younger adults (Kaede et al., 2016). Quinzi et al. reported increased lip strength and achieved clinical correction of atypical swallowing (Quinzi et al., 2020). Furthermore, Ingervall and Carlsson reported that lip‐closing training influences lip soft tissue formation in children with ILS, which increases the height of both lips and decreases the interlabial gap (Ingervall & Carlsson, 1982). Additionally, Yoshizawa et al.'s EMG study revealed that hypoxic lip‐closing training for the orbicularis oris muscles in adults with ILS decreases orbicularis oris muscle activity, despite the necessity of greater muscle activity to keep the lips closed due to ILS (Yoshizawa et al., 2018). Moreover, Takamoto et al. reported that lip‐closing training improves maximal lip closure force, exemplified by a shortened eating time, decreased food spill rates, and decreased daytime sleeping in older adults (Takamoto et al., 2018). Furthermore, lip‐closing training significantly increases the prefrontal cortical activity during lip closure (Takamoto et al., 2018). In contrast, the orbicularis oris muscle, directly associated with LCS, is one of the facial muscles controlled by the facial nerve (cranial nerve VII) (Shimada et al., 2001). Open–close mouth movements of fetuses in the womb are associated with developing facial muscles in the fetal stage (Gasser, 1967). The facial nerve develops at a relatively early fetal stage (after 27 gestational weeks) (Shimada et al., 2001). The facial nerve and the oral muscles that it controls are more developed than those around the nose and eyebrow during the fetal stage. Immediately after birth, neonates perform lip functions such as lip reflex and lip search reflex, which initiate breastfeeding. As facial growth progresses throughout development, lip function, including LCS, gradually increases (Fukami et al., 2010). ILS might inhibit healthy neural development in children. This study suggests that lip‐closing training effectively activates facial muscles and may activate the appropriate neuromuscular function in childhood.

Significant differences in the initial LCS among participants with abnormal habits and/or malocclusion were observed (Figure 5). Gamboa et al. demonstrated that higher EMG activity in subjects with ILS implies a higher muscular effort due to the need for lip sealing during functional activities (Gamboa et al., 2017). Children with a poor lip seal have larger overbite and overjet and lesser LCS during button pulling than those with a good lip seal (Yata et al., 2001). The presence of abnormal habits and/or malocclusion may directly lead to a lower LCS due to an association with oral dysfunction. It may be difficult to improve malocclusion solely by lip‐closing training if the malocclusion is induced by continuous and prolonged abnormal habits (Ingervall & Carlsson, 1982). Therefore, it is important to regularly evaluate oral function in children, recognize abnormal habits at an early stage, establish functionally sound oral habits in patients, and retain knowledge in their parents.

This study is significant as it clarifies the effects of lip‐closing training in childhood, for which there has been limited evidence. However, the study had several limitations. First, the home training employed might have been affected by the child's ability to remain focused during the exercise. Second, the absence of a control group that would have acted as the comparison group lowers the validity of our findings. The future objective is to clarify whether an increase in LCS improves ILS.

## 5 CONCLUSION

Lip‐closing training was carried out among 154 Japanese children aged 3–12 years with lower LCS due to ILS or abnormal habits using the Lipple‐trainer® at home for 3 months. All children showed greater LCS after lip‐closing training for short periods. The beginning of lip‐closing training was more effective than the subsequent periods. Anterior cross bite, including reverse bite, open bite, and tongue thrusting, significantly reduced training effects. The results suggest that less detrimental effects of malocclusion and abnormal oral habits lip‐closing training enhances LCS in younger children. LCS in children with ILS improved after lip‐closing training, but oral dysfunction inhibited the positive effects of training. The influence of ILS and malocclusion on each other, as well as the relationship between increased LCS and improved ILS in children, will be clarified in further studies.

## AUTHORS CONTRIBUTIONS

Yukiko Nogami, Issei Saitoh, Emi Inada and Yasutaka Kaihara conceived and designed the experiments; Yukiko Nogami, Issei Saitoh, Daisuke Murakami, Yoko Iwase, Naoko Kubota, Kuniko Nakakura‐Ohshima, Ayako Suzuki, and Yuki Nakamura performed the experiments; Issei Saitoh, Emi Inada and Yasutaka Kaihara analyzed the data; original draft preparation; Yukiko Nogami, Issei Saitoh, Youichi Yamasaki, Haruaki Hayasaki and Yasutaka Kaihara performed the writing–reviewing and editing; funding acquisition, Issei Saitoh, Emi Inada and Daisuke Murakami.

## CONFLICT OF INTEREST

The authors declare no conflict of interests.

## Data Availability

The datasets analyzed during the present study are not publicly available due to ethical restrictions 6 but are available from the corresponding author on reasonable request. All data analyzed during 7 this study are included in this published article.
